# Human fibroblast and stem cell resource from the Dominantly Inherited Alzheimer Network

**DOI:** 10.1186/s13195-018-0400-0

**Published:** 2018-07-25

**Authors:** Celeste M. Karch, Damián Hernández, Jen-Chyong Wang, Jacob Marsh, Alex W. Hewitt, Simon Hsu, Joanne Norton, Denise Levitch, Tamara Donahue, Wendy Sigurdson, Bernardino Ghetti, Martin Farlow, Jasmeer Chhatwal, Sarah Berman, Carlos Cruchaga, John C. Morris, Randall J. Bateman, the Dominantly Inherited Alzheimer Network (DIAN), Alice Pébay, Alison M. Goate

**Affiliations:** 1 Department of Psychiatry, Washington University School of Medicine, Campus Box 8134, 660 South Euclid Avenue, St. Louis, MO 63110 USA; 2 Centre for Eye Research Australia, Royal Victorian Eye and Ear Hospital, East Melbourne, VIC Australia; 3 Ophthalmology, Department of Surgery, University of Melbourne, Melbourne, VIC Australia; 4 Department of Neuroscience and Department of Genetics and Genomic Sciences, Ronald M. Loeb Center for Alzheimer’s Disease, Icahn School of Medicine at Mount Sinai, 1425 Madison Avenue, New York, NY 10029 USA; 5 School of Medicine, Menzies Institute for Medical Research, University of Tasmania, Hobart, Australia; 6 Department of Neurology, Washington University School of Medicine, 660 South Euclid Avenue, St. Louis, MO 63110 USA; 7 Department of Pathology and Laboratory Medicine, Indiana University, 635 Barnhill Drive, MS A 142, Indianapolis, IN 46202 USA; 8 Department of Neurology, Indiana University, 635 Barnhill Drive, MS A 142, Indianapolis, IN 46202 USA; 9 Massachusetts General Hospital, Athinoula A. Martinos Center for Biomedical Imaging, 149 13th Street, Charlestown, MA 02129 USA; 10 Alzheimer Disease Research Center, University of Pittsburgh School of Medicine, 4-West Montefiore University Hospital, 200 Lothrop Street, Pittsburgh, PA 15213 USA

**Keywords:** Dominantly Inherited Alzheimer Network, Amyloid precursor protein, Presenilin 1, Presenilin 2, Fibroblasts, Induced pluripotent stem cells

## Abstract

**Background:**

Mutations in amyloid precursor protein (*APP*), presenilin 1 (*PSEN1*) and presenilin 2 (*PSEN2*) cause autosomal dominant forms of Alzheimer disease (ADAD). More than 280 pathogenic mutations have been reported in *APP*, *PSEN1*, and *PSEN2*. However, understanding of the basic biological mechanisms that drive the disease are limited. The Dominantly Inherited Alzheimer Network (DIAN) is an international observational study of *APP*, *PSEN1*, and *PSEN2* mutation carriers with the goal of determining the sequence of changes in presymptomatic mutation carriers who are destined to develop Alzheimer disease.

**Results:**

We generated a library of 98 dermal fibroblast lines from 42 ADAD families enrolled in DIAN. We have reprogrammed a subset of the DIAN fibroblast lines into patient-specific induced pluripotent stem cell (iPSC) lines. These cells were thoroughly characterized for pluripotency markers.

**Conclusions:**

This library represents a comprehensive resource that can be used for disease modeling and the development of novel therapeutics.

**Electronic supplementary material:**

The online version of this article (10.1186/s13195-018-0400-0) contains supplementary material, which is available to authorized users.

## Background

Dominantly inherited mutations in amyloid precursor protein (*APP*), presenilin 1 (*PSEN1*), and presenilin 2 (*PSEN2*) cause early-onset Alzheimer disease (AD) [1, 2]. Sequential cleavage of APP, a type 1 transmembrane protein, by β-secretase and then by γ-secretase produces amyloid-β (Aβ) [3]. PSEN1 and PSEN2 are critical components of the γ-secretase complex. The amyloid cascade hypothesis proposes that changes in APP and/or Aβ homeostasis lead to the aggregation of Aβ and deposition in plaques and that these events are sufficient to initiate the cascade of pathologic abnormalities associated with AD [4]. In order to better understand AD, the Dominantly Inherited Alzheimer Network (DIAN) was established as an international effort to monitor and identify changes in *APP*, *PSEN1*, and *PSEN2* mutation carriers through the preclinical and clinical disease course. DIAN participants are monitored longitudinally with the goal of detecting and treating autosomal dominant Alzheimer disease (ADAD) at the earliest stages [5]. These efforts have begun to reveal fluid biomarker changes in ADAD mutation carriers as early as 20 years prior to the clinical onset of disease [5, 6].

Dominantly inherited mutations in *APP* account for approximately 16% of ADAD, represented by 30 pathogenic mutations [7]. Two recessive *APP* mutations, A673V and E693Δ, also reportedly cause AD (reviewed in [2]). The majority of mutations in *APP* cluster in exons 16 and 17, which encode the region that is adjacent to or within the Aβ domain. APP mutations impact Aβ production by several mechanisms: mutations adjacent to the α-secretase cleavage site lead to increased total Aβ, Aβ_40_, Aβ_42_, and Aβ_42/40_, whereas mutations near the γ-secretase cleavage site leads to reduced total Aβ and Aβ_40_ along with increased Aβ_42/40_ [8–10]. *APP* mutation carriers typically present with an age at onset ranging from 45 to 60 years [11]. *PSEN1* and *PSEN2* are structurally similar integral membrane proteins that contain nine transmembrane domains with a hydrophilic intracellular loop region (reviewed in [12]). *PSEN1* and *PSEN2* mutations are distributed throughout the protein, with some clustering occurring in the transmembrane domains [13]. PSEN1 and PSEN2 localize in the endoplasmic reticulum and Golgi apparatus, where they play an important role in protein processing [14, 15]. Mutations in *PSEN1* and *PSEN2* alter γ-secretase activity and exhibit an elevated Aβ_42/40_ ratio. As many as 185 dominantly inherited, pathogenic mutations have been identified in *PSEN1*, accounting for almost 80% of ADAD cases [7]. Individuals with *PSEN1* mutations present with the youngest and most variable ages at onset (between 30 and 75 years) [16]. To date, 13 dominantly inherited pathogenic mutations have been identified in *PSEN2*, which account for 6% of ADAD cases [7]. *PSEN2* mutation carriers exhibit the latest age at onset among ADAD mutations [16]. Although ADAD mutations are extremely rare, increasing evidence suggests that common variants in *APP*, *PSEN1*, and *PSEN2* may act as risk factors for AD [12, 17, 18].

Effective therapies have yet to be identified to modify or delay AD, which is due in part to the limitations of current cell and mouse models of AD. Most models rely on overexpression of a mutant transgene to study AD, which may produce effects that are a function of protein levels rather than a disease-relevant phenotype [19–21]. The majority of models capture amyloid or tau pathology but rarely both. Furthermore, although these models capture some secondary features of AD, such as gliosis, most do not produce frank neurodegeneration. With more than 280 pathogenic mutations across 3 genes, current model systems do not distinguish between those mechanisms that are shared among mutations and those that are unique. This is particularly critical when considering the potential impact of mutation status on responsiveness to therapies, such as γ-secretase modulators [22]. Thus, our understanding of how APP and tau are metabolized has been obtained from experimental paradigms that do not fully capture physiological conditions that are relevant to AD.

In this article, we present a resource of patient-specific fibroblast and induced pluripotent stem cell (iPSC) lines carrying *APP*, *PSEN1*, or *PSEN2* mutations and noncarrier, related controls. iPSCs have emerged as a powerful system for studying the molecular mechanisms underlying neurodegenerative diseases [23–27]. Human iPSCs express the regulatory elements that facilitate endogenous expression and splicing of genes associated with AD. Human iPSCs also have the capacity to be differentiated into cortical neurons or other cell types (e.g., glia) that are affected in AD [28–31]. Recent studies have shown that iPSC-derived neurons from *APP*, *PSEN1*, or *PSEN2* mutation carriers phenocopy aspects of the disease, including changes in Aβ isoform ratios and phosphorylated tau levels [22, 23, 28, 29, 32]. Advances in the generation of astrocyte, microglia, and cerebral organoids from iPSCs will facilitate future studies into the cell-autonomous and non-cell-autonomous effects of ADAD mutations [30, 31, 33, 34]. The iPSCs used in this study were obtained from individuals enrolled in DIAN, which collects neuropathological, clinical, imaging, biomarker (cerebrospinal fluid [CSF] and plasma), and genetic information that can be used to correlate with cellular phenotypes. Together, this resource represents a comprehensive resource for the broader scientific community to use to model AD and to develop novel therapeutics.

## Methods

### Patient consent

Skin biopsies were collected following written informed consent was obtained from the donor. The study was approved by the Washington University School of Medicine Institutional Review Board and Ethics Committee (IRB 201104178, 201306108). The consent allows use of tissue by all parties, commercial and academic, for the purposes of research but not for use in human therapy.

### Dermal fibroblast isolation

To isolate dermal fibroblasts, the skin biopsies were rinsed with PBS (MilliporeSigma, Burlington, MA, USA) and cut with dissecting scissors. The resulting tissue fragments were plated into a dry 24-well tissue culture plate. Excess PBS was removed, and fibroblast growth medium (Lonza, Basel, Switzerland) was added. Tissue was incubated at 37 °C, 5% CO_2_. After 24 hours, tissue was supplemented with fibroblast growth media, and media changes were repeated every 3–4 days. Fibroblast cells were observed to migrate from the tissue within 2 weeks of culture. Dermal fibroblasts were maintained in fibroblast growth media supplemented with penicillin and streptomycin. All fibroblasts are housed within the DIAN Genetics Core Tissue Bank and available for distribution upon request: https://dian.wustl.edu/our-research/observational-study/dian-observational-study-investigator-resources/biospecimen-request-terms-and-instructions/. Additional phenotype information is available upon request, including sex, age at biopsy, and other clinical, genetic, and biomarker measures collected within DIAN: https://dian.wustl.edu/our-research/observational-study/dian-observational-study-investigator-resources/data-request-form/.

### iPSC generation

Human fibroblasts were transduced with non-integrating Sendai virus carrying OCT3/4, SOX2, KLF4, and cMYC (Life Technologies, Carlsbad, CA, USA) in feeder- and serum-free conditions using mTesR (STEMCELL Technologies, Vancouver, BC, Canada) [35, 36]. Cells that showed morphological evidence of reprogramming were selected by manual dissection and maintained undifferentiated using mTesR1. A subset of *APP* fibroblast cell lines were reprogrammed into iPSCs using non-integrating episomal vectors expressing OCT4, SOX2, KLF4, L-MYC, LIN28, and p53 short hairpin RNA in feeder- and serum-free conditions using TeSR-E7 medium (STEMCELL Technologies) [37]. Subsequently, reprogrammed colonies were manually dissected to establish clonal cell lines for expansion and characterization. The reprogrammed cells were maintained undifferentiated using E8 medium (STEMCELL Technologies).

### iPSC characterization

Human iPSC lines were characterized using standard methods [35]. All lines were analyzed for pluripotency markers (OCT3/4, TRA-1-60), and a subset of lines were analyzed for additional markers (SOX2, NANOG, SSEA4) by immunocytochemistry and qPCR and for chromosomal abnormalities by karyotyping. Cell lines were confirmed to possess the appropriate genotype by Sanger sequencing. All iPSCs are housed within the DIAN Genetics Core Tissue Bank and available for distribution upon request along with clinical, genetic, and biomarker data collected in DIAN (*see* “Dermal fibroblast isolation” section for details).

### iPSC culture, banking, and quality control measures

Human iPSCs were cultured in mTesR1 on Matrigel-coated tissue culture-treated plates (Cultrex Basement Membrane Extract [BME]; Trevigen, Inc., Gaithersburg, MD, USA). For routine passaging and unless otherwise noted below, iPSCs were dissociated with Accutase (Innovative Cell Technologies, San Diego, CA, USA) for 3 minutes. Dissociated cells were collected in PBS and centrifuged at 750 rpm for 3 minutes. After medium was aspirated, a portion of the cells were plated on new Matrigel-coated plates in mTesR1. iPSCs were maintained with less than 5% spontaneous differentiation and were cryopreserved in mTesR supplemented with 10% dimethyl sulfoxide and 40% FBS. iPSCs are karyotyped every 20 passages to ensure clones maintain stable genomes. For the *APP* lines, iPSCs were cultured using Vitronectin XF in TeSR-E8 medium and passaged with ReLeSR (STEMCELL Technologies). All cell lines were confirmed to be mycoplasma-free using the MycoAlert mycoplasma detection kit (Lonza) according to the manufacturer’s instructions.

### Immunocytochemistry

Cells were washed and fixed with 4% paraformaldehyde (Sigma-Aldrich, St. Louis, MO, USA). Primary and secondary antibodies were diluted in 3% bovine serum albumin. The following antibodies were used (Life Technologies): SOX2, SSEA4, TRA-1-60, OCT-3/4, Alexa Fluor 594 donkey antirabbit, Alexa Fluor 488 goat antimouse, Alexa Fluor 488 donkey antirat, and Alexa Fluor 594 goat antimouse. Nuclei were counterstained with 4′,6-diamidino-2-phenylindole (DAPI; Life Technologies). Images were acquired on a Nikon Eclipse 80i fluorescence microscope (Nikon Instruments, Melville, NY, USA) using MetaMorph software (Molecular Devices, Sunnyvale, CA, USA). For the *APP* lines, immunocytochemistry was performed using the following primary antibodies: mouse anti-OCT3/4 (Santa Cruz Biotechnology, Dallas, TX, USA) and mouse anti-TRA-1-60 (MilliporeSigma). Cells were then immunostained with isotype-specific secondary antibodies (Alexa Fluor 568 or 488; Life Technologies). Nuclei were counterstained using Hoechst 33342 or DAPI (Sigma-Aldrich) and mounted in VECTASHIELD mounting medium (Vector Laboratories, Burlingame, CA, USA). Specificity of the staining was verified by the absence of staining in negative controls consisting of the appropriate negative control immunoglobulin fraction (Dako; Agilent Technologies, Santa Clara, CA, USA). Images were acquired on a Zeiss AxioImager M2 fluorescence microscope using ZEN software (Carl Zeiss Microscopy, Buffalo Grove, IL, USA).

### qPCR

RNA was extracted from cell pellets with an RNeasy kit (QIAGEN, Hilden, Germany), following the manufacturer’s protocol. Extracted RNA (10 μg) was converted to complementary DNA (cDNA) by PCR using the High-Capacity cDNA Reverse Transcriptase Kit (Life Technologies). Gene expression was measured in iPSCs using qPCR as previously described (*SOX2*, *POU5F1*, *LIN28A*, *NANOG*, *B3GALT5*, *PODXL*) [38]. Primers specific to Sendai virus (*SEV*) were included to evaluate the presence of virus remaining in the isolated clones. Primers specific to *GAPDH* were used as a control.

### Karyotyping

Chromosomal abnormalities were assessed by G-band karyotyping. For the *APP* lines, copy number variation (CNV) analysis of the original fibroblasts and iPSCs was performed using HumanCore BeadChip arrays (Illumina, San Diego, CA, USA). CNV analyses were performed using PennCNV [39] and QuantiSNP [40] with default parameter settings. Chromosomal aberrations were deemed to involve at least 20 contiguous single-nucleotide polymorphisms or a genomic region spanning at least 1 Mb [39–41]. The B allele frequency and the log R ratio were extracted from GenomeStudio (Illumina) for representation [42].

## Results

### Fibroblasts

**Fig. 1:**
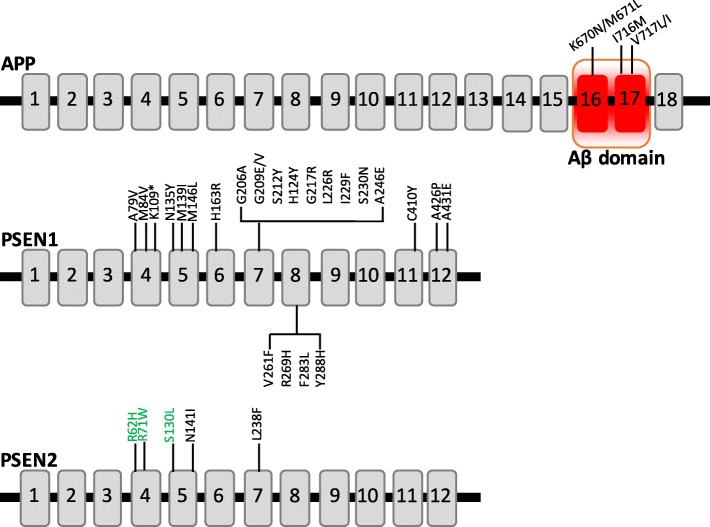
*APP*, *PSEN1*, and *PSEN2* mutations. Schematic of the location of *APP*, *PSEN1*, and *PSEN2* mutations reported in this collection. *Green* = variants of unknown pathogenicity. *Aβ* β-Amyloid

**Fig. 2:**
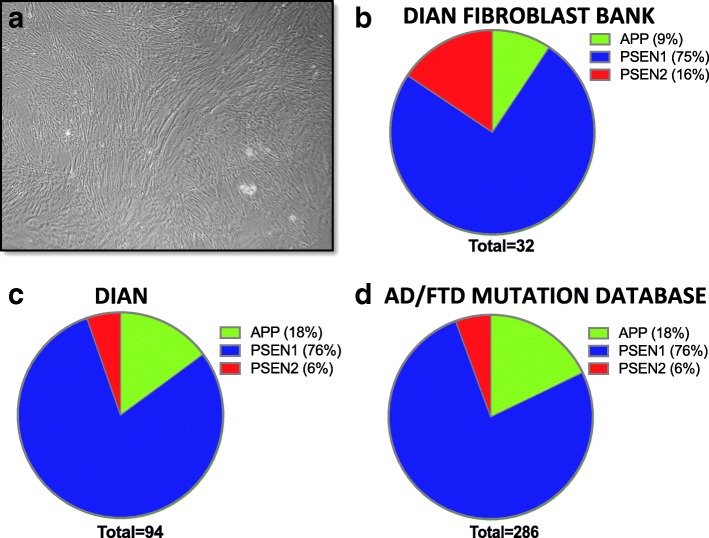
Dominantly Inherited Alzheimer Network (DIAN) fibroblast bank. **a** Representative bright-field image of human dermal fibroblasts. **b**–**d** Pie charts representing the percentage of *APP*, *PSEN1*, and *PSEN2* mutations represented in the DIAN fibroblast bank (**b**), DIAN observational study (**c**), and reported in the Alzheimer’s disease (AD)/frontotemporal dementia (FTD) mutation database (**d**) [7]

Dermal fibroblasts were collected from DIAN families at the Knight Alzheimer Disease Research Center at Washington University, Indiana University, University of Pittsburgh, and Brigham and Women’s Hospital. A total of 98 fibroblast lines are represented by 51 *APP*, *PSEN1*, or *PSEN2* mutation carriers and 47 non-carriers, related family members from 42 families (Fig. 1, Table 1). In order to maintain anonymity, cell lines are reported by family mutation, which may include mutation carriers or non-carriers. This fibroblast bank is representative of the relative proportion of mutations present in the DIAN observational study as well as those reported worldwide (Fig. 2) [7]. We are continuing to bank dermal fibroblasts from DIAN participants to expand the collection.

**Table 1:** Summary of fibroblast bank representing mutation carriers and noncarriers enrolled in the DIAN observational study

Gene	Mutation	Fibroblast lines	Families	Disease onset (yr)^a^	Disease course (yr)^a^	References
*APP*	KM670/671NL	3	1	52.3 ± 2.9	8.5 ± 3.5	[57]
	I716M	1	1	55	21	[58]
	V717 L	2	1	45.6 ± 1.2	9.33 ± 1.58	[57, 59–62]
	V717I	4	3	47.8 ± 0.9	11.86 ± 0.99	[57, 63–67]
*PSEN1*	A79V	11	4	60.6 ± 1.8	13 ± 1.86	[17, 57, 66, 68, 69]
	M84V	1	1	58.6 ± 1.36	20	[70]
	K109*	1	1	39	17	N/A
	N135Y	1	1	35.5 ± 4.5	9 ± 1	N/A
	M139I	4	1	35.6 ± 0.6	7.75 ± 1.97	[57, 71]
	M146L	1	1	39.3 ± 0.9	5.88 ± 1.09	[57, 59, 72–74]
	H163R	7	3	46.1 ± 0.7	12.14 ± 1.3	[57, 75–77]
	G206A	3	2	55.2 ± 1.3	11.6 ± 2.18	[57, 78]
	G209E	1	1	53.25 ± 4.40	4.75 ± 0.48	[79]
	G209V	1	1	52	10	[77]
	S212Y	1	1	45.3 ± 2.2	14.5 ± 2.11	[57, 80]
	H214Y	1	1	52.67 ± 2.67	9 ± 2	[52, 81]
	G217R	3	1	44.6 ± 0.9	12.18 ± 2.19	[57, 82]
	L226R	4	1	46.7 ± 1.8	8.67 ± 1.2	[57]
	I229F	2	1	40 ± 2.1	18	[57]
	S230N	1	1	57.3 ± 1.45	6.50 ± 2.5	N/A
	A246E	2	1	49.1 ± 1.1	13.17 ± 2.63	[57, 83]
	V261F	1	1	34 ± 1.2	15 ± 2	[57]
	R269H	2	1	56.4 ± 2	10 ± 1	[57, 83]
	F283L	3	1	41	11	N/A
	Y288H	5	1	45.7 ± 1.7	17	[57]
	C410Y	2	1	47.7 ± 1.1	9.88 ± 2.46	[57, 83]
	A426P	1	1	43.36 ± 1.38	13.71 ± 1.11	[77]
	A431E	1	1	39.4 ± 0.6	9 ± 0.86	[57, 84, 85]
*PSEN2*	R62H^b^	1	1	63.5 ± 15.5	12	[13, 52, 54, 86]
	R71W^b^	2	1	66.50 ± 18.50	18.0 ± 10	[13, 87]
	S130L^b^	1	1	50.50 ± 3.5	18.50 ± 6.5	[52–55]
	N141I	23	2	53.7 ± 0.6	10.23 ± 0.44	[57, 88, 89]
	L238F	1	1	53 ± 4	20	[70]

*N/A* Not available

^a^Mean ± SE

^b^Pathogenicity unclear

*stop

### Generation and characterization of iPSCs

**Fig. 3:**
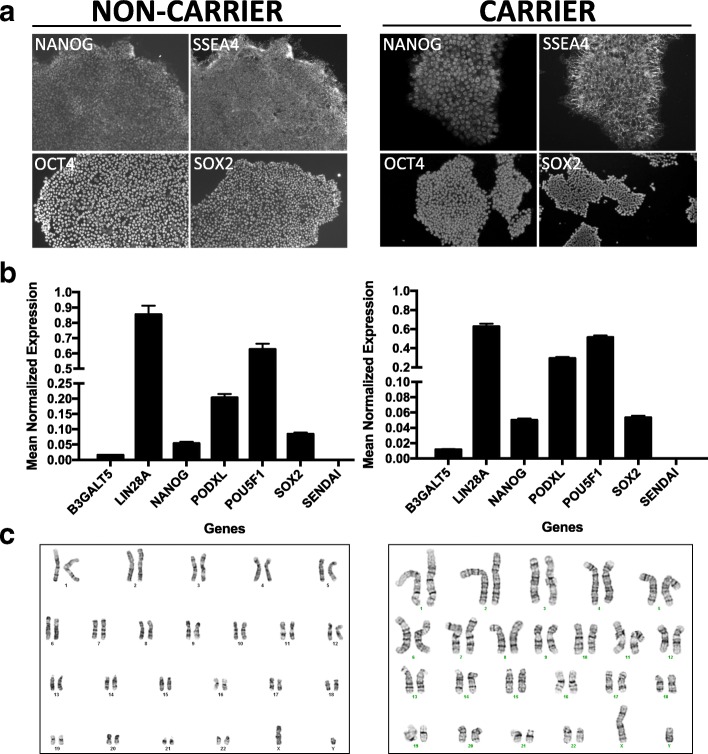
Characterization of Dominantly Inherited Alzheimer Network (DIAN) induced pluripotent stem cell (iPSC) lines. Representative images of non-mutation carrier (*left panel*) and mutation carrier (*right panel*) iPSCs. **a** Immunostaining for pluripotency markers NANOG, SSEA4, OCT-3/4, and SOX2. **b** qPCR for pluripotency markers. **c** Karyotyping

iPSCs were generated using non-integrating Sendai virus or episomal vectors (Table 2). iPSCs were grown in feeder-free and serum-free conditions. Resulting iPSCs have been characterized for pluripotency (Fig. 3; Additional file 1: Figures S1-S3 and Additional file 2: Figure S4). Pluripotency was defined on the basis of morphology and markers of pluripotency expression by immunocytochemistry and qPCR (Fig. 3b and c; Additional file 1: Figures S1 and S2). Endogenous expression of pluripotency markers was evaluated relative to dermal fibroblasts and H9, an embryonic stem cell line. Some variability was observed in the pluripotency markers between individual donor lines (Additional file 1: Figures S1 and S2). This is consistent with prior reports that genomic background is the largest contributor to phenotypic variability between iPSC lines [41]. We confirmed the silencing of exogenous Sendai virus-driven pluripotent markers by qPCR (Fig. 3c; Additional file 1: Figure S2). Chromosomal stability was assessed by G-band or digital karyotyping (Fig. 3d; Additional files 1 and 2: Figures S3 and S4). iPSC lines meeting the following criteria are available upon request: (1) maintain pluripotency with less than 5% spontaneous differentiation; (2) OCT4- and TRA1-positive by immunostaining; (3) endogenous expression of *LIN28A*, *NANOG*, *PODXL*, *POU5F1*, and *SOX2* as measured by qPCR; (4) absence of Sendai virus and Sendai-driven genes as measured by qPCR; (5) absence of chromosomal abnormalities as measured by G-band or virtual karyotyping (CNV analysis); and (6) the ability of the iPSC clones to differentiate into neurons. All of the iPSC lines included in the collection meet the above-mentioned criteria. We are continuing to reprogram dermal fibroblasts to expand the stem cell bank.

**Table 2:** Human induced pluripotent stem cells from mutation carriers and non-carriers enrolled in DIAN

Gene	Family mutation	Mutation status	Donors^a^	Ethnicity	Clinical status^b^	APOE	Reprogramming method	Donor number
*APP*	V717L	Positive	1	EA	A	33	Episomal	F15553
	V717I	Positive	1	EA	A	33	Episomal	F16574
		Negative	1	EA	A	33	Episomal	F12462
*PSEN1*	A79V	Positive	1	EA	A	34	Sendai	F12424
		Negative	1	EA	A	33	Sendai	F12436
	H163R	Positive	1	EA	A	34	Sendai	F11430
		Negative	1	EA	A	33	Sendai	F12442
	G217R	Positive	1	EA	A	24	Sendai	F12434
		Negative	1	EA	A	34	Sendai	F12445
*PSEN2*	N141I	Positive	1	EA	A	33	Sendai	F12448
		Negative	1	EA	A	34	Sendai	F12468

*APOE* Apolipoprotein E, *EA* European American

^a^Independent induced pluripotent stem cell lines. For each line, at least two clones are available

^b^At biopsy

## Discussion

Rare mutations in *APP*, *PSEN1*, and *PSEN2* cause ADAD; however, the mechanisms by which altered APP processing leads to changes in tau and cognitive decline remain poorly understood. DIAN was established in 2008 to recruit families that carry *APP, PSEN1*, or *PSEN2* mutations. Observational studies in these families have demonstrated that biomarker changes can occur 15–20 years prior to the estimated age at onset of AD [6, 43]. We established a resource of patient-specific fibroblast and iPSC lines carrying *APP*, *PSEN1*, or *PSEN2* mutations and non-carrier, related controls. The human cell lines are associated with neuropathological, clinical, imaging, biomarker (CSF and plasma), and genetic information that can be used to correlate with cellular phenotypes.

The DIAN fibroblast bank represents cell lines from the most common ADAD mutations. Several of the mutations are represented by multiple families, including *APP* V717I, *PSEN1* A79V, *PSEN1* H163R, *PSEN1* G206A, and *PSEN2* N141I. Fibroblasts from ADAD mutation carriers produce altered levels of extracellular Aβ_42_, which are further exaggerated in neural progenitor cells and cortical neurons [23]. Additionally, recent work demonstrates that direct conversion of fibroblasts into cells of neuronal fate maintains epigenetic signatures associated with aging that are lost when differentiation occurs through iPSCs [44, 45]. With 98 fibroblast lines from 42 ADAD families, this resource offers the opportunity to identify phenotypes that are shared across families as well as those phenotypes that may be unique to a given family, possibly due to disease-modifying factors in the genetic background. Additionally, some mutations are represented by more than ten donor lines, such as *PSEN1* A79V and *PSEN2* N141I, which will allow for the investigation of cellular and molecular modifiers of age at onset within a family.

We generated a subset of iPSCs from mutations that are representative of ADAD. *APP* V717I (e.g., London mutation), which is represented in the iPSC bank, has been reported in 38 families [46]. Introduction of the isoleucine at this site results in an elevated Aβ_42/40_ ratio. *APP* V717I presents with amyloid plaques, neurofibrillary tangles, cerebral amyloid angiopathy [47], and, in some cases, with amygdala Lewy bodies [48]. iPSC-derived neurons expressing *APP* V717I produce altered APP processing and tau expression [29]. Several pathogenic mutations have been reported at amino acid 717 in APP. *APP* V717L has been reported in seven families and, similarly to the London mutation, produces an elevated Aβ_42/40_ ratio. *PSEN1* A79V, H163R, and G217R present with amyloid plaques, neurofibrillary tangles, and variable amygdala Lewy bodies [48]. *PSEN2* N141I presents with amyloid plaques, neurofibrillary tangles, and amygdala Lewy bodies [48]. iPSC-derived neurons from *PSEN1* H163R and *PSEN2* N141I carriers also exhibit altered Aβ_42/40_ [22, 49].

Several fibroblast lines in the DIAN collection are derived from families carrying variants in *PSEN2* that have unclear pathogenicity: R62H, R71W, and S130L. *PSEN2* is known to be highly polymorphic. *PSEN2* R62H has been reported in seven families; however, little segregation data is available [7]. *PSEN2* R62H is present in a large cohort of unselected controls (Exome Variant Server: 187/12819; ExAC Browser: 1198/121044). Given the frequency in control populations, this variant is likely benign. In two families, *PSEN2* R71W segregates with disease [50] and has been reported in sporadic AD [13, 51, 52]. *PSEN2* R71W is present in a large cohort of unselected control subjects (Exome Variant Server: 36/12970 alleles; ExAC Browser: 407/121230 alleles); however, lack of clinical information and age precludes the determination of whether these variant carriers have preclinical AD. Given that this variant has been reported in patients with AD, it is possible that *PSEN2* R71W increases risk for AD. *PSEN2* S130L has been reported in one family with a strong history of ADAD [53]. However, *PSEN2* S130L has also been identified in two control subjects and several patients with sporadic AD [52, 54, 55]. In an unselected control population, *PSEN2* S130L is also present (Exome Variant Server: 9/12997 alleles; ExAC Browser: 77/119594 alleles). In cell culture, *PSEN2* S130L expression alters the Aβ_42/40_ ratio [56]. Thus, *PSEN2* S130L may modify disease risk. Together, these lines will facilitate the study of disease-modifying variants in AD.

## Conclusions

The field has struggled to move drugs and potential druggable targets from mouse models into effective therapies. This may be due in part to model systems that do not fully capture APP and tau metabolism in human cell types that are affected in disease. The fibroblast and iPSC resource that we report represents a unique opportunity to translate findings from cells to the human subjects from whom they were obtained.

## Additional files

Additional file 1: Characterization of iPSC lines. **Figure S1.** Immunostaining of DIAN iPSCs for pluripotency markers. iPSCs included in the collection were fixed and stained with antibodies to OCT4 and TRA1. Scale bar represents 100 μm. **Figure S2.** Quantitative assessment of pluripotent markers in DIAN iPSCs. iPSCs lines were analyzed by qPCR (TaqMan assay) to determine expression of pluripotency markers and, in lines reprogrammed with Sendai virus, the absence of Sendai virus. Human embryonic stem cells (H9) were included as a positive control. Genes are expressed relative to a housekeeping gene, *GAPDH*. Graphs represent mean normalized expressed ± SEM. **Figure S3.** Karyotypes of DIAN iPSCs. G-band karyotyping of iPSCs exhibit no chromosomal abnormalities in the clones represented in the collection. (PDF 12885 kb)

Additional file 2: Virtual karyotyping of iPSC lines. **Figure S4.** Virtual karyotyping. (PDF 102140 kb)
