# Supplementary material for: Human fibroblast and stem cell resource from the Dominantly Inherited Alzheimer Network
Source: Alzheimers Res Ther. 2018 Jul 25;10:69. doi: 10.1186/s13195-018-0400-0 (PMC6060509; doi:10.1186/s13195-018-0400-0)
Supplement: Supplementary file 1 — Characterization of iPSC lines. Figure S1. Immunostaining of DIAN iPSCs for pluripotency markers. iPSCs included in the collection were fixed and stained with antibodies to OCT4 and TRA1. Scale bar represents 100 μm. Figure S2. Quantitative assessment of pluripotent markers in DIAN iPSCs. iPSCs lines were analyzed by qPCR (TaqMan assay) to determine expression of pluripotency markers and, in lines reprogrammed with Sendai virus, the absence of Sendai virus. Human embryonic stem cells (H9) were included as a positive control. Genes are expressed relative to a housekeeping gene, GAPDH. Graphs represent mean normalized expressed ± SEM. Figure S3. Karyotypes of DIAN iPSCs. G-band karyotyping of iPSCs exhibit no chromosomal abnormalities in the clones represented in the collection. (PDF 12885 kb) [file 13195_2018_400_MOESM1_ESM.pdf]

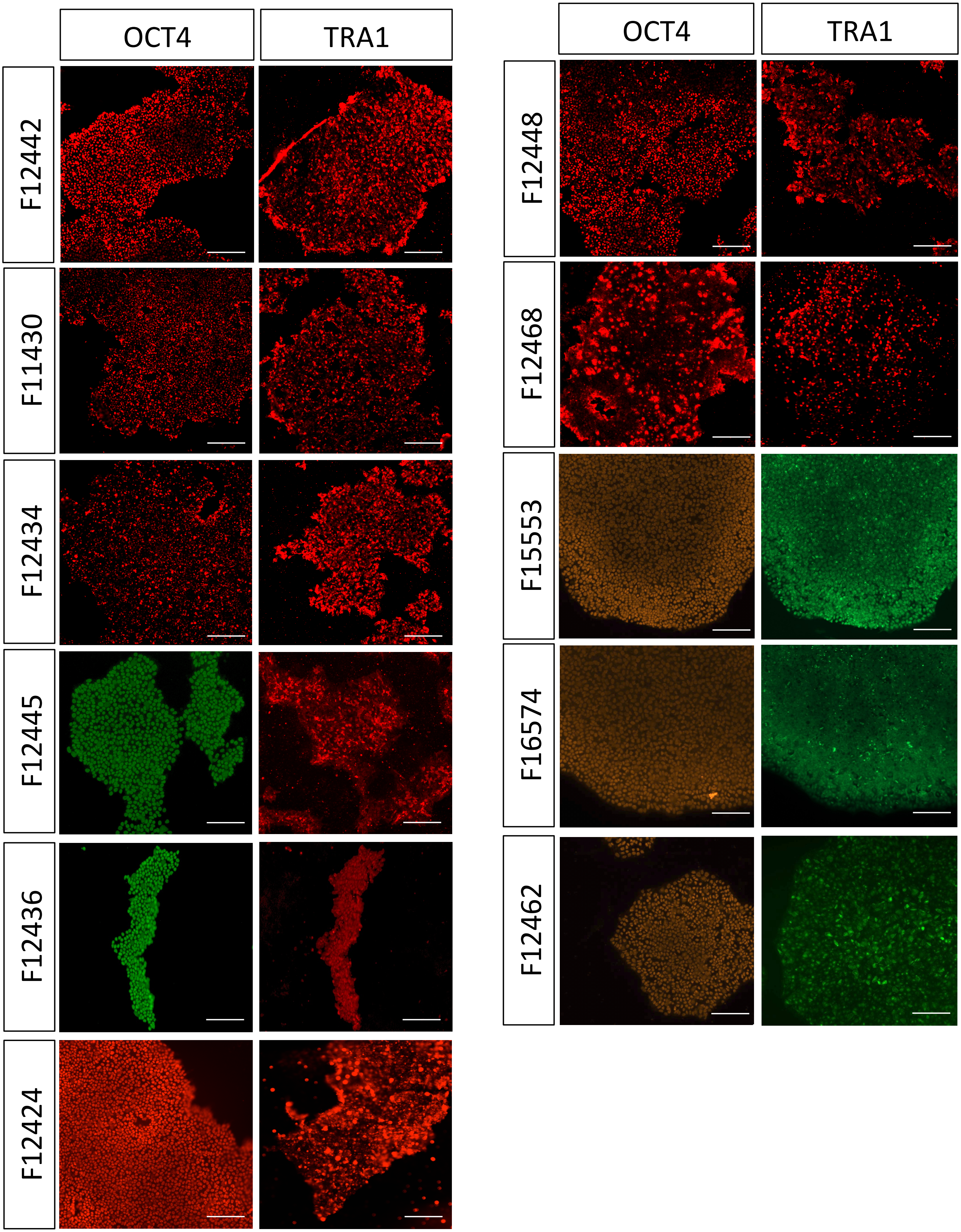


**Supplemental Figure 1: Immunostaining of DIAN iPSC for pluripotency markers.** iPSCs included in the collection were fixed and stained with antibodies to OCT4 and TRA1. Scale bar represents 100µM.

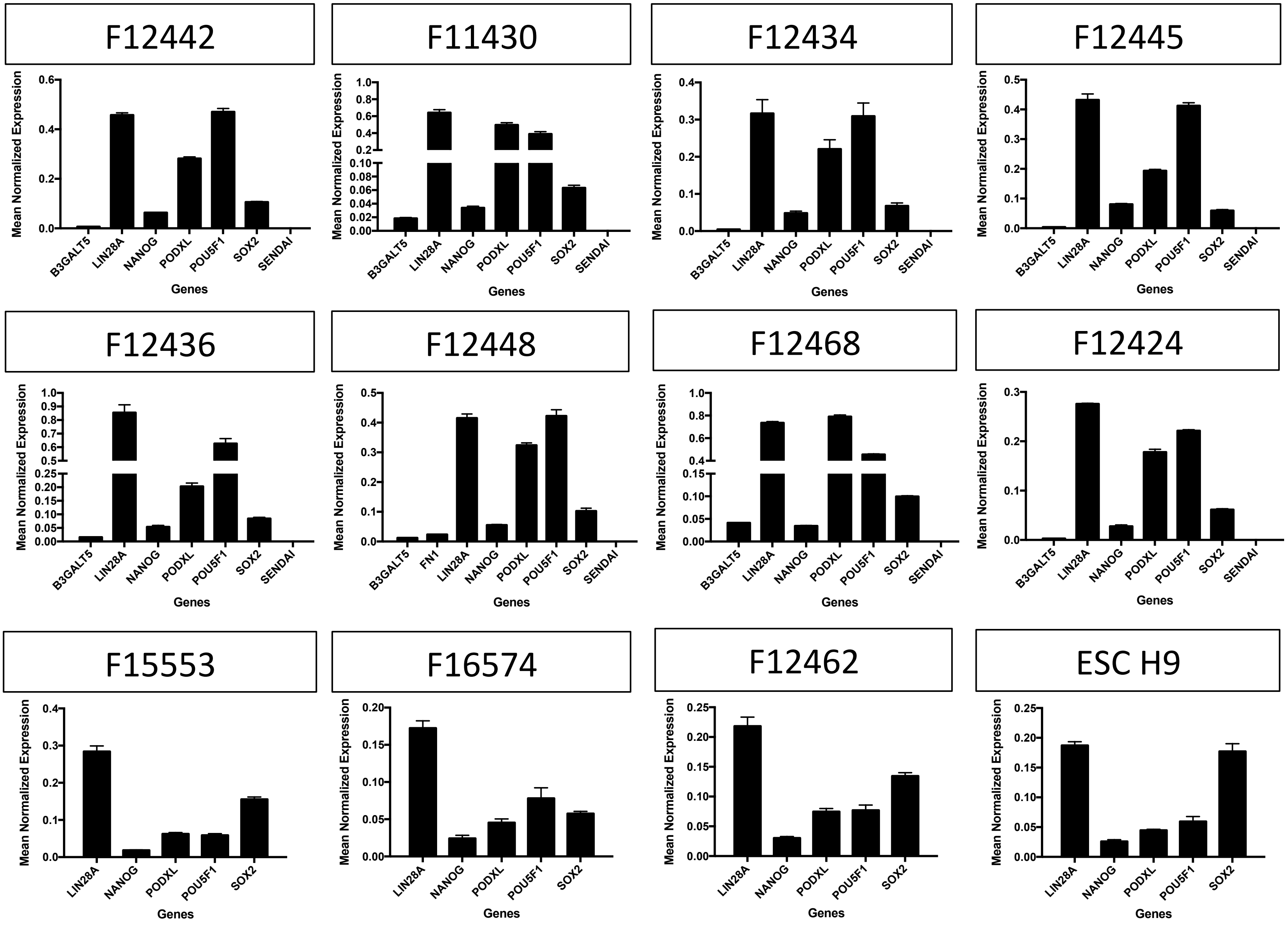


**Supplemental Figure 2: Quantitative assessment of pluripotent markers in DIAN iPSC.** IPSC lines were analyzed by qPCR (Taqman assay) to determine expression of pluripotency markers and, in lines reprogrammed with Sendai virus, the absence of Sendai virus. Human embryonic stem cell (H9) were included as a positive control. Genes expressed relative to a housekeeping gene, *GAPDH*. Graphs represent mean normalized expressed ± SEM.

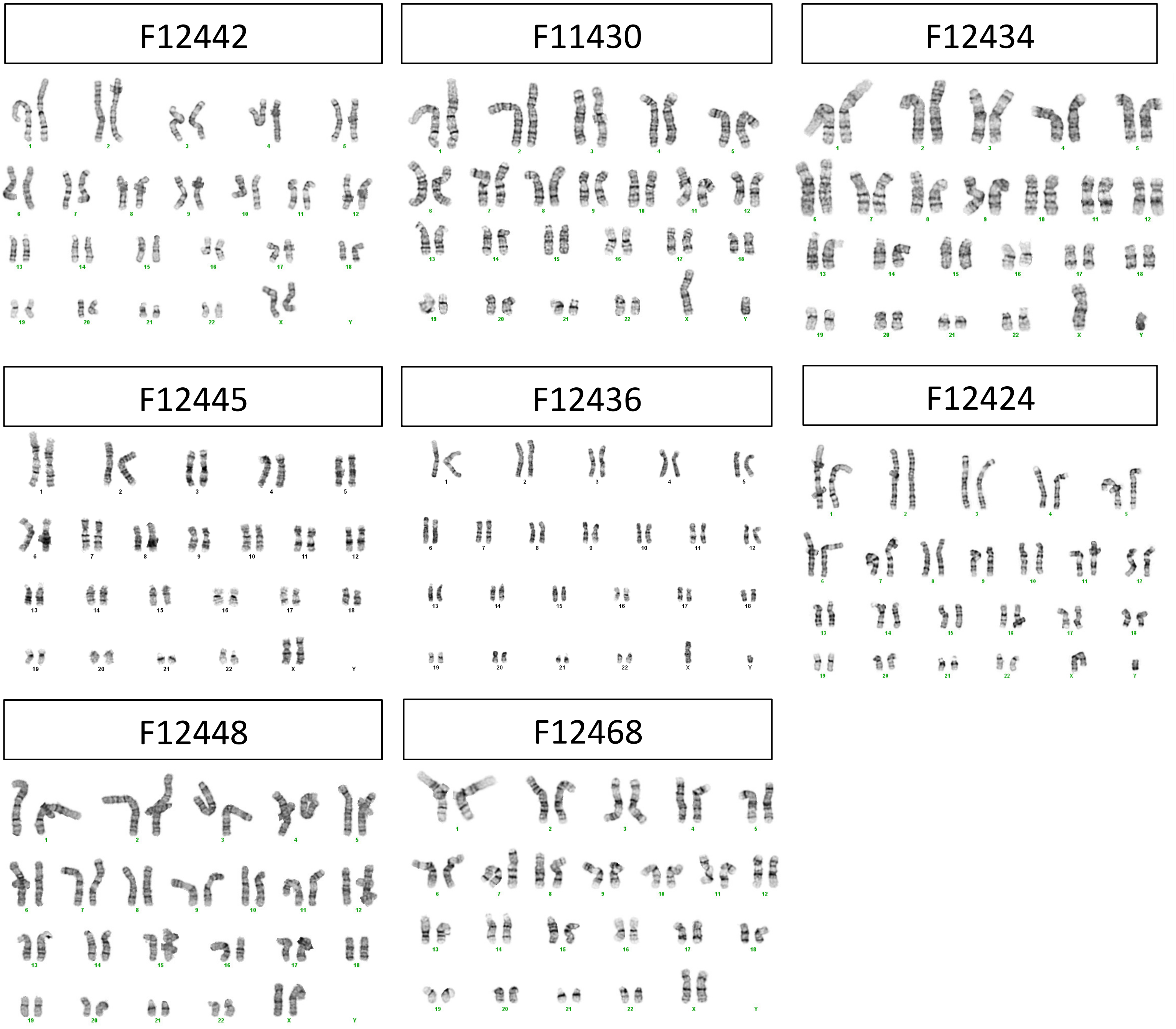


**Supplemental Figure 3: Karyotypes of DIAN iPSCs.** G-band karyotyping of iPSC exhibit no chromosomal abnormalities in the clones represented in the collection.
