# Supplementary material for: Human fibroblast and stem cell resource from the Dominantly Inherited Alzheimer Network
Source: Alzheimers Res Ther. 2018 Jul 25;10:69. doi: 10.1186/s13195-018-0400-0 (PMC6060509; doi:10.1186/s13195-018-0400-0)
Supplement: Supplementary file 2 — Virtual karyotyping of iPSC lines. Figure S4. Virtual karyotyping. (PDF 102140 kb) [file 13195_2018_400_MOESM2_ESM.pdf]

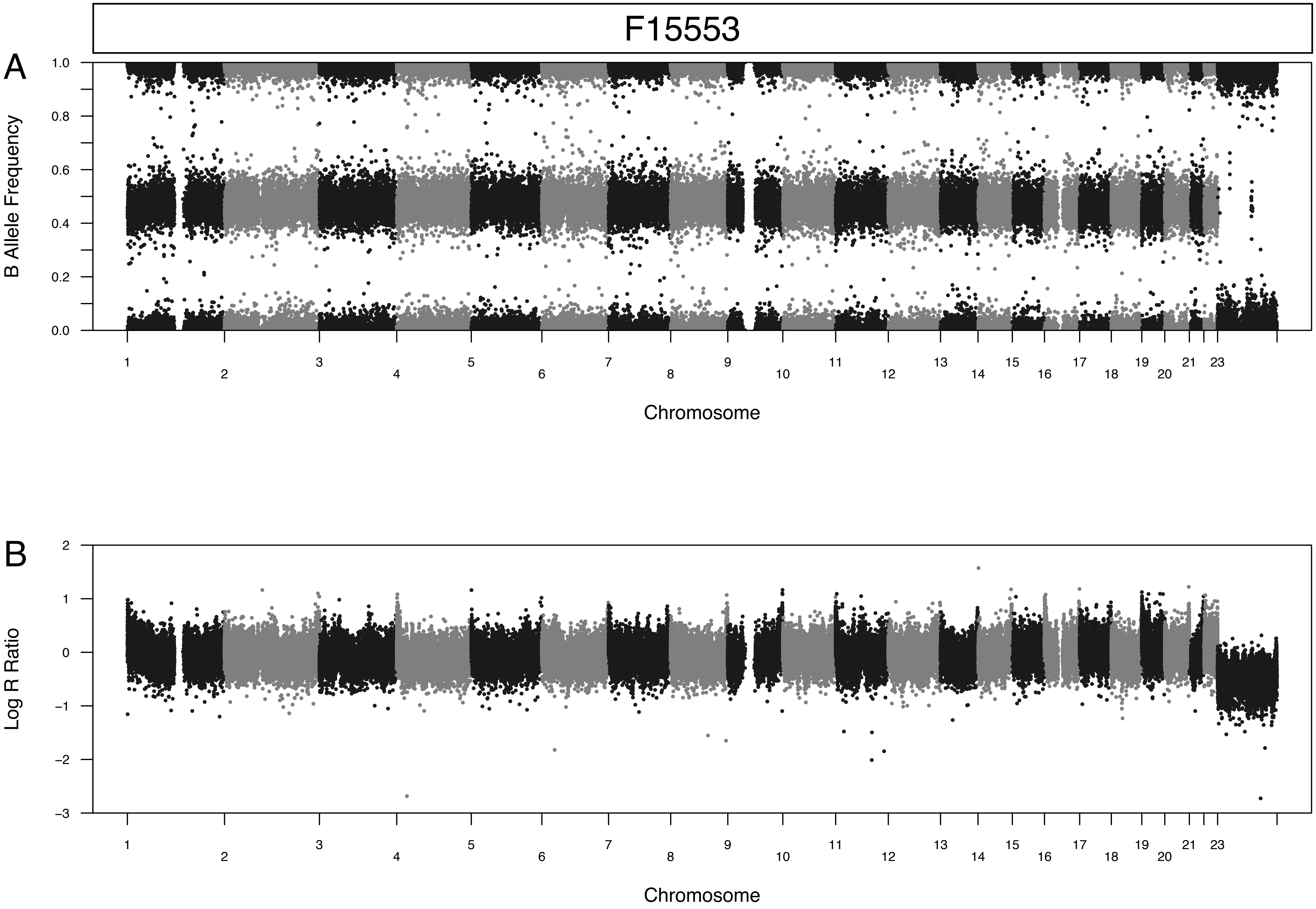
F15553
A
B Allele Frequency
1.0
0.8
0.6
0.4
0.2
0.0
1
2
3
4
5
6
7
8
9
10
11
12
13
14
15
16
17
18
19
20
21
23
Chromosome
B
Log R Ratio
2
1
0
−1
−2
−3
1
2
3
4
5
6
7
8
9
10
11
12
13
14
15
16
17
18
19
20
21
23
Chromosome

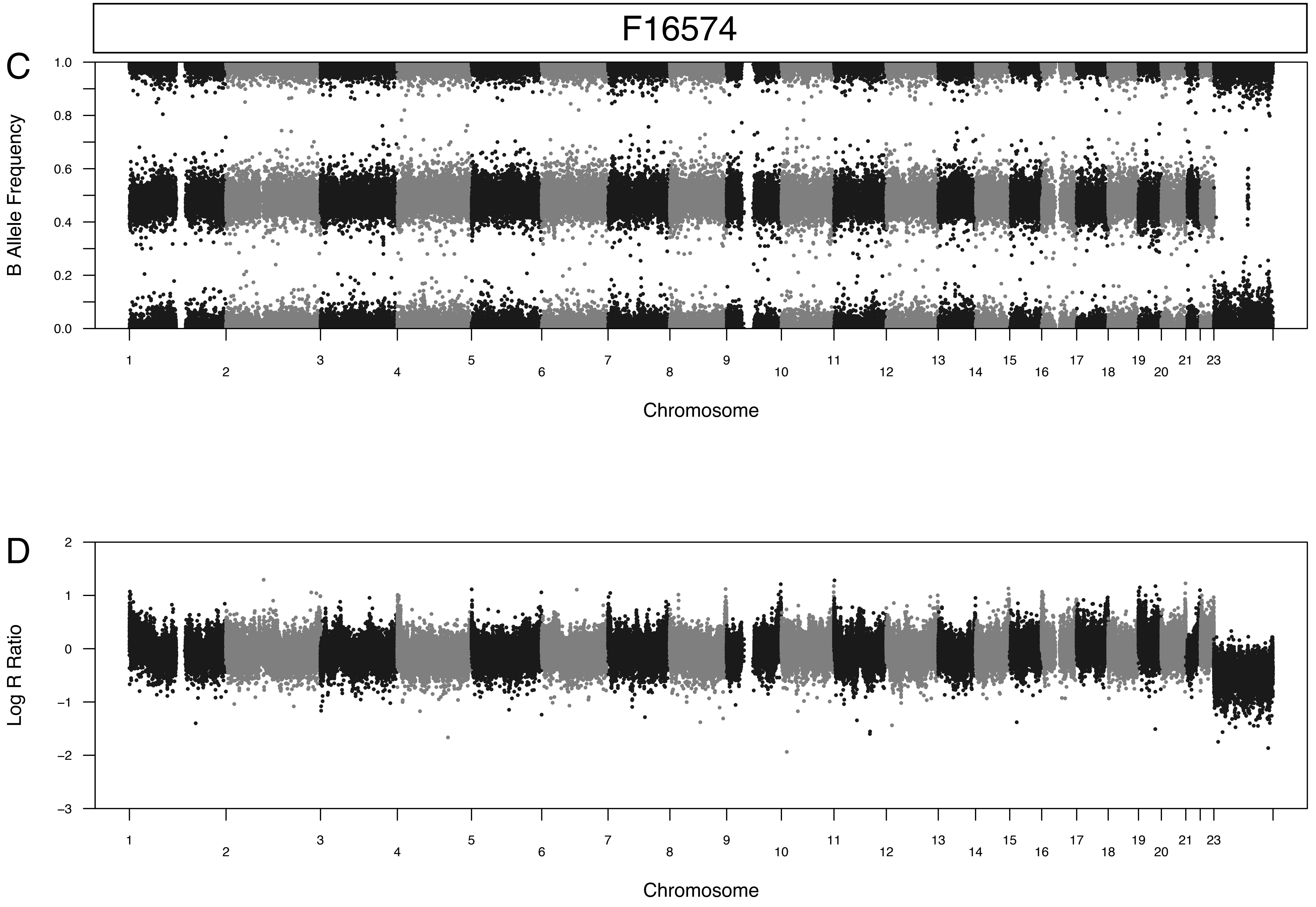
F16574
C
B Allele Frequency
0.0
0.2
0.4
0.6
0.8
1.0
Chromosome
1
2
3
4
5
6
7
8
9
10
11
12
13
14
15
16
17
18
19
20
21
23
D
Log R Ratio
−3
−2
−1
0
1
2
Chromosome

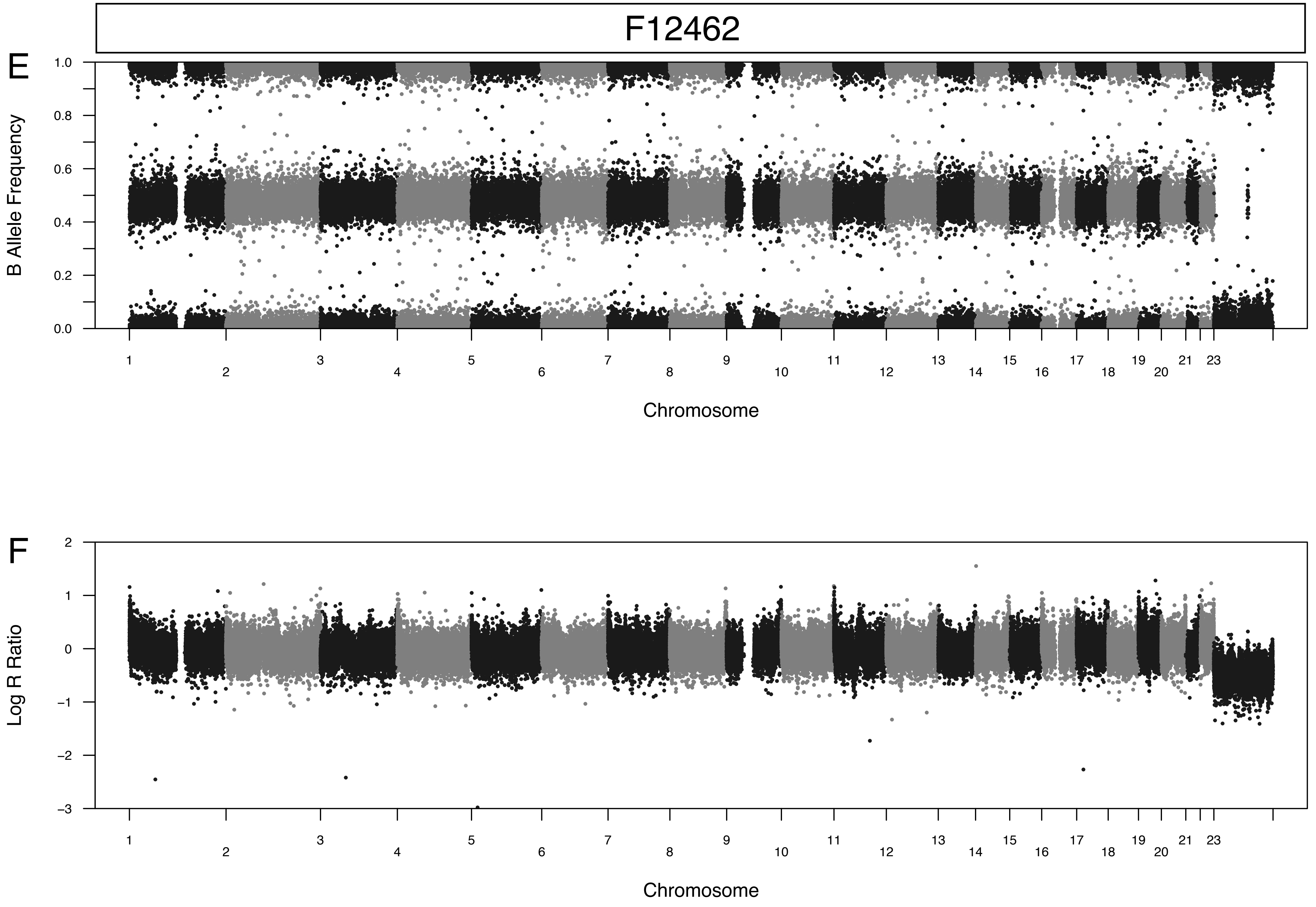

F12462
E
B Allele Frequency
Chromosome
F
Log R Ratio
Chromosome

Supplemental Figure 4: Virtual karyotyping of a subset of DIAN iPSC. A-B. F15553. C-D. F16574. E-F. F12462. A, C, E. B Allele Frequency. B, D, F. Log R Ratio.
